# Understanding the Effects of Age and T-Cell Differentiation on COVID-19 Severity: Implicating a Fas/FasL-mediated Feed-Forward Controller of T-Cell Differentiation

**DOI:** 10.3389/fimmu.2022.853606

**Published:** 2022-03-03

**Authors:** Anthony J. Leonardi, Christos P. Argyropoulos, Adam Hamdy, Rui B. Proenca

**Affiliations:** ^1^Environmental Health and Engineering, Johns Hopkins Bloomberg School of Public Health, Baltimore, MD, United States; ^2^Department of Internal Medicine, Division of Nephrology, University of New Mexico School of Medicine, Albuquerque, NM, United States; ^3^Independent Researcher, Port Louis, Mauritius; ^4^Department of Biology, Johns Hopkins University, Baltimore, MD, United States

**Keywords:** Fas, CD8, T cells, differentiation, paracrine, FasL, SARS-CoV-2, feed-forward Loop

## Introduction

Feed-forward loops are means of managing homeostasis under dynamic conditions (1–4). It is a way for organisms and cells to manage inputs and responses while maintaining optimal functioning (1).

Feed-forward control differs from feedback control in a number of ways, including how a feed-forward controller is 1) responsive to process parameter changes in the system and 2) corrects for disturbances in the system (4). While the more widely known feedback systems track a continuous measurement (“an error signal”) and attempt to correct it by matching their “control signals”, feed-forward systems are command and control: once the control signal has been issued, it cannot be adjusted until a new control signal has been issued.

An example will make the distinction between these two forms of control clearer. While driving, slowing down before making a turn is a form of feed forward control, because the driver (“controller”) anticipates the effects of inertia upon the moving car and attempts to pre-compensate. Adaptive cruise control offers an example of a feedback system: the car’s “computers” continuously measures the speed of the car and either accelerates or decelerates to keep the speed constant at the desired level. In this analogy, the Fas and FasL expression induced following stimulation (5) are the ‘control’ signals, and the ensuing differentiation, cytotoxic acquisition, and apoptosis *via* Fas (which can terminate in effector function and death) is the ‘turning’ maneuver. The maneuver (turning vs. differentiation with death or effector function) has a preparation stage that depends on the ‘speed’ (which is the differentiation state of the T-cells in the pool).

Feed-forward loops (FFLs) are common in physiological systems and immunological responses (1, 2, 6, 7). For example, the mTORC1/Akt axis has been shown to be regulated in a feed-forward manner, and a feed-forward inflammatory loop has been implicated in lethal influenza (7, 8). Notably, the Akt pathway has been shown to be the predominant pathway controlling T cell memory and effector differentiation (9). Illustrating its importance for T-cell effector function, the inhibition of this pathway following stimulation uncouples T cell differentiation and proliferation following T cell stimulation and keeps t cells in the memory state (10, 11).

T cells can exert a contact-dependent paracrine Fas-FasL mediated differentiation effect on co-stimulated T cells *in vivo* and *in vitro (*5, 12, 13) with directionality from the FasL-expressing memory and effector cells to the Fas-expressing naïve T cells and other memory/effector cells. Following stimulation but before differentiation occurs, a non-apoptotic Fas (CD95) signal travels through the Akt pathway in order to differentiate T-cells. Illustrating this, Fas blockade in a pool of T cells following stimulation *in vitro* also uncouples differentiation and proliferation of T cells and prevents their terminal effector differentiation and release of effector cytokines. This means the canonical death receptor CD95 is involved in the function and fate of T cells in a pool. This behavior of T-T interactions which influence the acquisition of effector function represents quorum decision-making for differentiation, cytotoxicity, and apoptosis, all *via* Fas, implying T cells behave as a highly regulated quorum sensing tissue that relies on its homotypic immediate environment for context (14).

**Figure 1:**
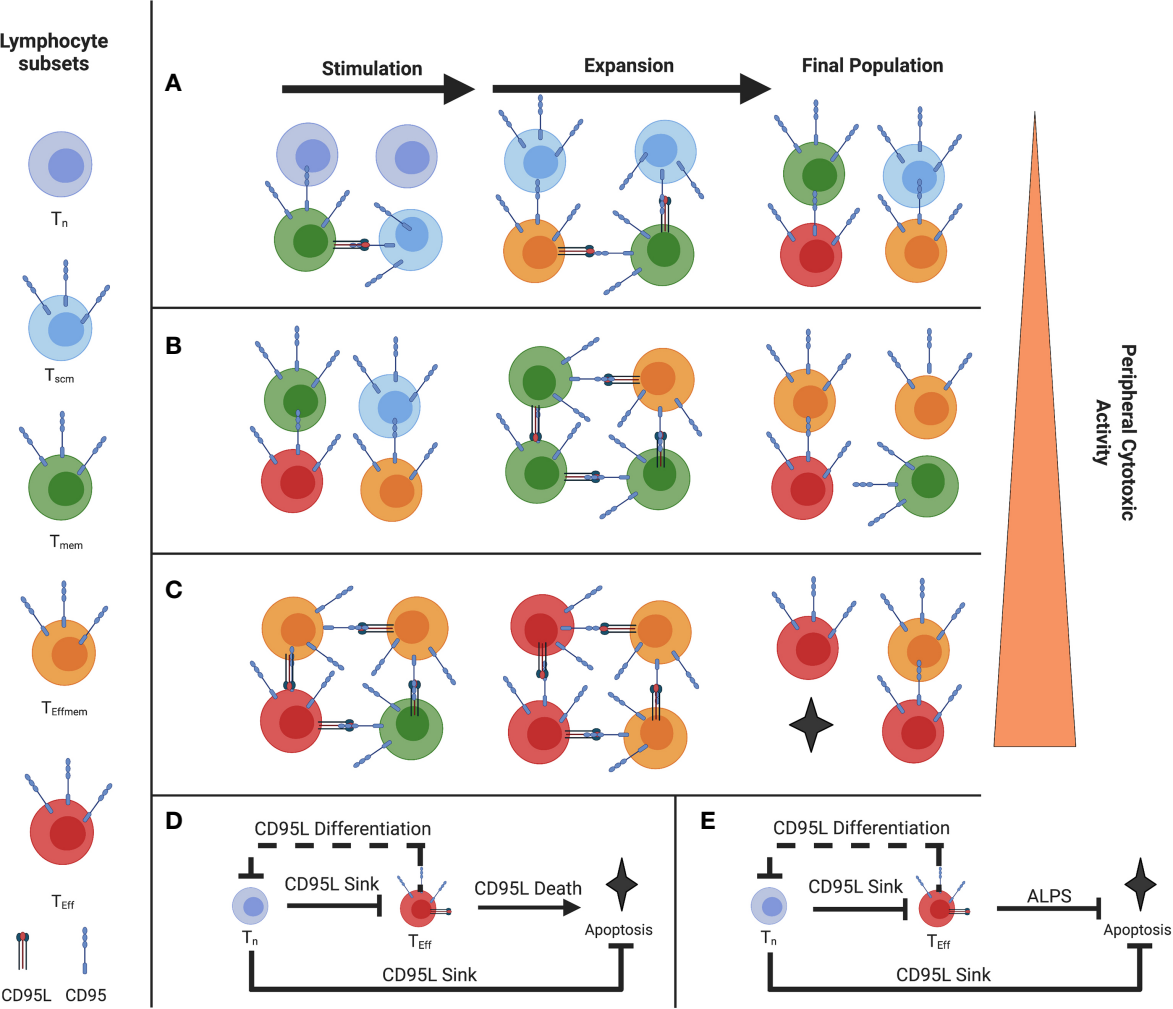
Illustration of the Paracrine Fas/FasL differentiation and death system **(A)** Stimulation of a naive-rich pool of T-cells corresponding to a youthful repertoire **(B)** Stimulation of a more differentiated pool of T-cells **(C)** Stimulation of a pool of T-cells with few Tn, where the population will reach cytotoxic function more exuberantly **(D)** Proposed coherent type 2 Feed-Forward Loop of Fas-mediated differentiation and death (solid line), with a negative feedback loop on naive T cells from effector T cells **(E)** FFL error from coherent type 2 to incoherent type 2 in ALPS and the murine model Fas C194V due to a defect in CD95-mediated Apoptosis but not CD95-mediated differentiation. (Figure adapted with permission from Leonardi and Proenca, 2020).

Typically, feed-forward control is described in a single cell, but since T cells are able to cause the differentiation of paracrine T cells and also ‘soak up’ like a cytokine sink or withhold differentiation of paracrine T cells, they are able to act as a quorum (**Figures 1A–C**) (5). In this sense, a single cell with a given phenotype, such as an activated naive or Tscm CD8 T cell expressing CD95 for example, is able to inhibit effector differentiation of adjacent memory cells by ‘soaking up’ or acting as a ‘sink’ for CD95L, for example.

That is the first component of the feed-forward loop we describe in **Figure 1D**. The second component of the loop are the effector cells (in red) that highly express both CD95 and CD95L and will agonize adjacent cells and bring about their differentiation and subsequent death, which is limited to terminally differentiated effectors (5). By terminating the FFL with apoptosis in those cells which are terminally differentiated and therefore have high effector function, this allows for self-limiting effector responses.

However, the naive and Tscm cells will prevent this from occurring when they are present, by virtue of ‘soaking up’ this CD95L signal (5). The sum of this interaction comprises a feed-forward loop (**Figure 1D**). This interaction we believe is a method in control in the balance of T cell homeostasis between the blood, lymphoid organs, and peripheral tissues.

In lethal cases of Covid-19 there is a necrosis of the spleen and lymph nodes perhaps indicative of excessive T cell effector differentiation (15, 16). Indeed, many peripherally-infiltrating lymphocytes in infection are short-lived and are not accompanied by Naive and Tscm cells that could prevent their full effector function (17). Furthermore, immune privileged sites like the eye exploit this susceptibility by expressing FasL to quickly delete wayward T cells (18, 19). Therefore, this Terminal portion of the Fas-FasL feed-forward loop is an essential component of immune tolerance and T cell control (20, 21).

The specific kinetic and dynamic mirrors a type 2 coherent Feed Forward Loop where disturbances are corrected for and responses are accelerated and then quite literally, terminated, by apoptosis (2, 3, 5, 6). Prior evidence includes 1) how naive CD8 T-cells act as a “sink”, withholding differentiation and cytotoxic function in memory CD8 T-cells (5), and 2) how acute T-T interactions can prime Tumor-specific effector T cells to more exuberantly act against tumor (22).

The significance of this Feed-Forward Loop is shown by the protective effect of Tn CD8+ cells in Covid-19 (23), which reconciles the highly discrepant outcomes seen between children (low mortality) (24, 25) and elderly adults (high mortality) (26, 27). A Fas-mediated role of excessive T-cell death in severe cases has recently become more evident as well, lending credibility to the earlier proposal about its role in apoptosis (12, 28).

Indeed, André et al. propose blocking Fas-mediated death *via* a pan caspase inhibitor Q-VD (28). Finally, given the differentiation and depreciation of the Tn pool from infection likely due to the overshooting T-cell stimulation from SARS-Cov-2’s cryptic (29) accumulation of superantigenic biomass (12, 30, 31), T cell activation *via* complement (32), and bystander activation (33), we can use the model to anticipate the possibility of worsening disease upon reinfection in cases where there is not adequate early control and a highly differentiated T cell repertoire (34). This does not mean all reinfections will be worse, rather that they will be worse when the parameters of this feed-forward model are met.

## Discussion

### What Explains Heightened Severity in the Aged and Mild Illness in the Youth?

There is a dire need to explain the wide gamut of immune dysfunction in Covid-19 between mild and severe disease (35). Here, we incorporate a major correlation of Covid-1 severity; the proportion of CD8+ naïve T cells (12, 23, 36). As described by Moderbacher, et al., a higher proportion of these cells is protective in Covid-19. Indeed, much of the pathology in Covid-19 is T cell driven, and it would be useful to incorporate these observations into a system/model that reconciles them. We previously described how Tn and Tscm can act as ‘sinks’ for a differentiation and death signal (12, 37). de Candia et al. proposed that naive repertoires exert better control by virtue of greater TCR repertoire diversity which historically is associated with lower CD4 and CD8 T cell activation (38). however no model reconciles 1) the low Case Fatality Rate (CFR) seen in children (24, 25); 2) the high CFR seen in the elderly (39); 3) cases of more severe reinfection (40); 4) the efficacy of steroids, which cause a rapid reduction in CD8+ T-cells, and suppress Fas and FasL in T-cells (41–45); 5) the efficacy of anti GM-CSF (46, 47), which downregulates Fas expression on T-cells (48); and the high degree of Fas-mediated T cell apoptosis which characterizes severe Covid-19 (28).

### Proposing A Fas/Akt Differentiation and Death Feed-Forward Loop

We previously described a system where, under costimulation, Tscm expressed CD95 and acted as a ‘sink’ for CD95L expressed by Teff, Tem, and Tcm (12, 37). This CD95L expression induces differentiation in proximal cells where CD95 is ligated, except in terminally-differentiated cells where it induces death (5). This effect can be blocked with CD178 blockade or AKT inhibition without affecting T cell proliferation (5, 12). Based on the differentiation status/proportion of Tn of the T cell population undergoing stimulation, we can gauge how prone to exuberant effector function the resultant pool of T cells will behave, with higher proportion of Tn being protective as described by Moderbacher et al. (23) (**Figures 1A–C**) As FasL is expressed later in the stages of T cell differentiation, there is an epigenetic controller regulating its expression as part of a canonically developmental type 2 coherent feed forward loop (2, 3, 5) (**Figure 1D**). Furthermore, there is negative feedback in the system where Tem push the differentiation of CD95-expressing Tn (or Tscm) which reduces the dampening effect of the Tscm by reducing their numbers and promoting their acquisition of CD95L. This negative feedback is prototypically implemented alongside feed-forward loops (4, 49). The FFL ends when CD8+ T cells that have reached terminal differentiation undergo apoptosis and are removed from the pool. Disturbances in this terminal element of coherent FFL control are exemplified in ALPS, where the apoptotic function of Fas is ablated but the terminal effector differentiation is maintained (50) (**Figure 1E**). Indeed, we see the consequence of what is ideally, a type 2 *coherent* FFL mutagenically switched to a type 2 *incoherent* FFL which loses the termination of the differentiation effect *via* apoptosis, as described by Mangon and Alon (2, 49). The difference in whether a FFL is coherent vs. incoherent depends on the sign (positive or negative, respectively) of the output (2). For example, in the condition ALPS Fas- mediated death is disrupted, so the FFL drives to terminal effector differentiation without apoptosis, reversing the sign of the outcome (2, 21). As such, we can contextualize the autoimmune toxicity to peripheral tissues and lymphoproliferation and see the physiological utility of the coherent FFL as opposed to an incoherent FFL in the Fas/Akt Differentiation and Death feed-forward system (2, 5, 12, 37, 50, 51).

### Perilous “Recall”: Depletion Dependent Enhancement

In SARS Cov-2 infection, peripheral blood CD8+ phenotypes exhibit a significant reduction in the proportion of Tn and an increase of Teff (52). In a single cell analysis of T cell memory following infection, Adamo et al. observe that severe cases of Covid-19 are marked by a CD8+ T cell effector population they assume is induced by bystander activation, and mild cases marked by a memory phenotype (53). Cohen et al. examined the differences in T cell induction of IFN-γ and found increased age was associated with higher T-cell activation and concluded that reduced the T-cell activation in children may be responsible for milder Covid-19 (25). Previously, we described a model of T-cell driven Covid-19 severity incorporating the finding of Moderbacher, et al., where an increased proportion of CD8+ Tn had a protective effect (12, 23, 37). In the model, we showed T cells with a higher proportion of Fas expression would differentiate due to a Fas-potentiated non-apoptotic Akt signal, thereby causing the exuberant T cell effector function seen in severe cases (12). Given the possible frequency of Covid-19 reinfection and rechallenge, the model can be updated to incorporate T cell differentiation and effector function conferred by reinfection. Based upon data showing a retraction of the Tn repertoire following infection by Townsend et al, and Phetsouphanh et al, we can speculate regarding a possible “T cell depletion dependent enhancement” as the stimulated repertoire (which includes the T cells specific to SARS-Cov-2 epitopes, those activated by a bystander effect, and those also stimulated superantigenically and by complement) has lost a proportion of its Tn and is thereby able to quickly differentiate into T cell Effectors since there is no CD95L sink that the CD95 expression on Tn and Tscm offer (30–32, 54). This effect would be more evident in the aged 60+, who have significant reductions in CD8+ Tn proportions following SARS Cov-2 infection, due to the bystander, complement, and superantigen-induced excessive stimulation (30–32, 34). Of course, primed T-cells such as those from vaccination are capable of exerting earlier control of infection (55); and indeed, early CD8 bystander activation is associated with better control of infection (33), so we anticipate a degree of protection conferred from vaccination when the individual has a paucity of naïve T cells, such as in **Figure 1C**. We must note, however, infection with SARS-CoV-2 represents a challenge for the CD8+ compartment given its immune evasion, like Major Histocompatibility Complex 1 (MHC 1) downregulation (29). In cases where early control is not accomplished, T cell memory could contribute to an overexuberant response (12, 37, 38). Indeed, exuberant CD8+ T-cell mediated pathology has been documented in infections like RSV and SARS-CoV-2 alike (32, 56, 57). Memory CD8+ responses have been shown to exert immunopathology and severe disease in murine models of RSV (56).

Additionally, this model suggests a pathogenic role in FasL-mediated T cell differentiation in the development of Type 1 diabetes, as shown in the NOD mice model by Xiao, et al. (58, 59) In NOD mice, CD8+ T-cells have been shown to exert β-cell destruction, which can be abrogated with FasL blockade, which would reduce CD8+ effector differentiation and function (11, 58, 59). This pathway is growing in relevance considering the risk for newly diagnosed autoimmune diabetes (Type 1) following SARS-Cov-2 infection, and consideration of this model and the nonspecific T-cell activation and differentiation observed in COVID-19 would proffer mechanism in part (60).

### Pride Goeth Before The Fall: The Utility of Apoptosis Following Differentiation

On T-cells, the CD95 receptor potentiates a differentiation signal *via* AKT until the T-cell reaches terminal differentiation, whereby CD95 engagement induces apoptosis (11, 50). Evolutionarily, programming apoptosis into the same system of effector differentiation by the adaptive immune system is an elegant means of assuring self-limiting effector T cell responses. Where CD95-mediated death does not occur, such as in cases of ALPS, T-cell effector populations may accumulate, manifesting in autoimmunity and lymphoproliferation (61, 62).

### “Driven” by Danger, Effector T Cells Cut the Brake Lines and Step on the Gas

The feed-forward model proposed here represents a quorum-like T cell dynamic in activated states (63). To revisit our analogy, naïve T cells are like the ‘brakes’ for a high response whereby they ‘absorb’ CD95L. In situations with persistent and broad activation of naïve T cells they would be subjected to FasL expressing cells upon arrival to the lymph nodes and differentiated by those means. Long Covid’s paucity of naïve T cells would fit this dynamic (54). As shown in the dotted negative feedback line in **Figure 1D**, FasL-expressing effectors can precociously differentiate naïve T cells, like “cutting the brakes” before a turn (12, 37). Such negative feedback loops are canonically found alongside feed-forward loops (4, 49). If there was a persistent or evasive antigenic reservoir capable of evading adaptive immunity and continually stimulating T cells it could explain the persistent elevation of Effectors and depletion of Tn seen in some cases of Long Covid (54). Persistent activation could hasten T cell differentiation and manifest in a naïve depletion, effectively manifesting as “cutting the brakes” in this model where naïve T cells dampen exuberant responses. A persistent command on the feed-forward controller could deplete the naive T cell subset by this mechanism, if the stimulation is broad enough. This mechanism may also, in part, describe an insidious lymphocyte depletion where there is chronic activation and loss of T populations in chronic infections with substantial bystander activation (64).

## Conclusion

T-cell differentiation and acquisition of effector function is accomplished *via* Feed-forward control. In Covid-19, there is nonspecific and possibly bystander cytotoxic CD8+ T-cell activation which may be a double-edged sword, exerting damage to tissues and vital organs like the lung and pancreas (14, 30–32, 52, 53, 60). The FFL proposed here gives a mechanism for both the exuberant T-cell response observed in severe cases and the protective effect of Tn (12, 23, 32, 37). It also anticipates the consequences of a diminishing pool of Tn if we are to consider the documented reduction of the naive T cell repertoire in SARS Cov-2 convalescence (34, 52), which may be induced by the superantigenic nature of infection and the bystander activation of T cells (30, 32). If this dynamic is correct and appreciable, COVID-19 reinfections may manifest more severe disease as T cell repertoires age and Tn reduce in frequency, manifesting in an individual and demographic level.

## Author Contributions

AL contrived the model and co-wrote the manuscript with CA, AH, and RP. All authors contributed to the article and approved the submitted version.

## Conflict of Interest

The authors declare that the research was conducted in the absence of any commercial or financial relationships that could be construed as a potential conflict of interest.

## Publisher’s Note

All claims expressed in this article are solely those of the authors and do not necessarily represent those of their affiliated organizations, or those of the publisher, the editors and the reviewers. Any product that may be evaluated in this article, or claim that may be made by its manufacturer, is not guaranteed or endorsed by the publisher.
